# Explainable machine-learning model for postoperative lower-extremity deep vein thrombosis after gynecologic cancer surgery

**DOI:** 10.3389/fonc.2026.1813354

**Published:** 2026-09-30

**Authors:** Yanhong Zhang, Congcong Shi, Deyan Yang, Xiaona Ji, Yingli Chu

**Affiliations:** 1 Department of Interventional Therapy I, Shandong Cancer Hospital and Institute, Shandong First Medical University and Shandong Academy of Medical Sciences, Jinan, Shandong, China; 2 Depression Disorder Diagnosis and Treatment Center, Shandong Mental Health Center, Jinan, Shandong, China; 3 Ward 1, Lung Surgery, Shandong Cancer Hospital and Institute, Jinan, Shandong, China

**Keywords:** CatBoost, deep vein thrombosis, explainable artificial intelligence, gynecologic oncology, machine learning, perioperative nursing, Shapley additive explanations (SHAP), thromboprophylaxis

## Abstract

**Background:**

Postoperative lower-extremity deep vein thrombosis (DVT) is a common and potentially fatal complication after radical surgery for gynecologic malignancies, yet population-specific risk tools remain limited.

**Methods:**

We retrospectively analyzed 500 consecutive patients who underwent radical surgery for cervical, endometrial, or ovarian cancer (January 2022-January 2025). The cohort was randomly divided into a training set (n=351, 70%) and a validation set (n=149, 30%) at a 7:3 ratio. Independent predictors were identified using logistic regression, and 10 machine-learning algorithms were trained and compared. Model discrimination, calibration, and clinical utility were evaluated, and the optimal model was interpreted using SHAP.

**Results:**

DVT occurred in 116 patients (23.2%). Five variables were independently associated with DVT: age, Caprini score, lymphocyte count, preoperative D-dimer, and neutrophil-to-lymphocyte ratio (NLR).Across candidate models, CatBoost achieved the optimal overall performance on the validation set, yielding an AUC of 0.816 (95% CI: 0.738–0.881), with sensitivity of 0.824 (95% CI: 0.700–1.000) and specificity of 0.730 (95% CI: 0.515–0.876), and SHAP analysis ranked the predictors by mean absolute SHAP value in the following order: lymphocyte count, Caprini score, age, preoperative D-dimer, and neutrophil-to-lymphocyte ratio (NLR).

**Conclusion:**

An interpretable CatBoost-based model enables individualized postoperative DVT risk stratification after gynecologic cancer surgery and may support targeted, evidence-based thromboprophylaxis and perioperative nursing management.

## Introduction

Gynecologic malignancies, including cervical, endometrial, and ovarian cancers, remain a major health burden for women. In China, national cancer statistics reported approximately 290,000 new gynecologic cancer cases and 100,000 related deaths in 2022 (1). Radical surgery is a cornerstone of treatment but is accompanied by a high risk of venous thromboembolism, particularly lower-extremity DVT, with reported postoperative incidences of 19.6%-38% (2). Undetected DVT can progress to pulmonary embolism and substantially increase morbidity and mortality (3, 4).

Several risk assessment tools are used in routine practice (e.g., Caprini and Autar), but their performance is inconsistent in gynecologic oncology because they are not tailored to tumor-related hypercoagulability, perioperative factors, and inflammatory dysregulation (5). Machine learning (ML) can model complex, non-linear relationships in multidimensional clinical data and has been increasingly applied to prediction tasks in oncology and perioperative care (6–11).

A key barrier to clinical translation is the perceived ‘black-box’ nature of many ML algorithms. To address interpretability and support bedside adoption, we used SHapley Additive exPlanations (SHAP), a game-theory-based approach that quantifies each feature’s contribution to model output at both global and individual levels. We aimed to develop and validate an interpretable ML model to predict postoperative lower-extremity DVT in patients undergoing radical surgery for gynecologic malignancies, compare performance across 10 algorithms, and identify clinically actionable predictors to inform precision thromboprophylaxis and perioperative nursing strategies.

## Materials and methods

This retrospective cohort study included patients who underwent radical surgery for histopathologically confirmed cervical, endometrial, or ovarian cancer at Shandong Cancer Hospital and Institute between January 2022 and January 2025. All eligible patients had preoperative laboratory testing and standardized lower-extremity duplex ultrasonography at admission and within 1 month postoperatively or at discharge to screen for DVT. The study was conducted in accordance with institutional policies and relevant regulations; details of ethics approval and consent procedures should be provided in the Ethics Statement section.For perioperative thromboprophylaxis, institutional standardized protocols were followed for all surgical patients. Briefly, low molecular weight heparin based pharmacological prophylaxis was administered for patients without bleeding related contraindications. Mechanical prophylaxis, including intermittent pneumatic compression devices and graduated compression stockings, was also routinely applied. Most pelvic oncologic radical surgeries were performed in the lithotomy position.

### Study design and participants

Inclusion criteria were: (i) age >=18 years; (ii) histopathologic diagnosis of cervical, endometrial, or ovarian cancer according to standard criteria (12); (iii) availability of preoperative laboratory results within 1 week before initial treatment; and (iv) complete perioperative records.

Exclusion criteria were: (i) pre-existing lower-extremity DVT; (ii) acute or chronic inflammatory conditions (e.g., pulmonary infection or autoimmune disease); (iii) anticoagulant and/or antiplatelet therapy within 1 month before admission; and (iv) psychiatric comorbidity involving substance dependence/addiction.

### DVT definition

Postoperative DVT was diagnosed using bilateral lower-extremity venous duplex ultrasonography following the Chinese Medical Association guideline (13). The common femoral, deep femoral, superficial femoral, popliteal, anterior tibial, posterior tibial, and peroneal veins were examined. Diagnostic criteria included venous incompressibility, intraluminal echogenic thrombus, filling defects, and abnormal flow augmentation or attenuation.

### Data collection

Data were extracted from the electronic medical record system and covered: (i) demographics and clinical variables (age, body mass index, comorbidities, postoperative bed rest duration, Caprini score, histological type and tumor stage, operative time, and intraoperative blood loss); (ii) laboratory variables (albumin, D-dimer, fasting glucose, activated partial thromboplastin time, prothrombin time, fibrinogen, platelet count, neutrophil count, monocyte count, lymphocyte count, high-density lipoprotein, and derived inflammatory indices including NLR, PLR, LMR, and MHR); and (iii) surgical variables (approach, operative duration, and blood loss).

### Data preprocessing

Continuous variables were retained in their original scale. Categorical variables were one-hot encoded. Missing data were handled by complete-case analysis, and 4 patients were excluded due to missing values. The dataset was randomly split into a training set (70%, n=351) and an independent validation set (30%, n=149). Features with wide numerical ranges were scaled using min-max normalization, with scaling parameters fitted solely on the training set to avoid data leakage.

### Feature selection

To derive a clinically parsimonious feature set, univariate comparisons were first performed only in the training set between the DVT and non-DVT groups. Variables with P<0.05 were entered into a multivariable binary logistic regression model to identify independent predictors while controlling for potential confounding. All feature selection procedures were conducted within the training set to eliminate data leakage.

### Model development

Ten distinct machine learning algorithms were trained on the training set: logistic regression (LR), support vector machine (SVM), gradient boosting machine (GBM), neural network (NN), random forest (RF), extreme gradient boosting (XGBoost), k-nearest neighbors (k-NN), adaptive boosting (AdaBoost), light gradient boosting machine (LightGBM), and categorical boosting (CatBoost). Hyperparameter optimization for each algorithm was performed using grid search with predefined parameter grids, allowing systematic evaluation of candidate hyperparameter combinations.

### Model evaluation and interpretability

Discrimination was quantified using the area under the receiver operating characteristic curve (AUC). Accuracy, sensitivity, specificity, precision, and F1-score were also calculated. Calibration was assessed using calibration curves, the calibration intercept, calibration slope, Hosmer–Lemeshow test, and Brier score. Decision curve analysis was performed to evaluate the clinical utility of the model. The DeLong test was used to compare AUC differences among different models. Bootstrap internal validation with 1000 resamples and optimism correction was applied to examine model stability and avoid overfitting. The optimal model was interpreted using SHAP analysis, including global feature importance and summary plots, as well as individual force plots.

### Statistical analysis

Statistical analyses were performed using SPSS (version 27.0) and R software (version 4.3.1). Normally distributed continuous variables were presented as the mean ± standard deviation (SD) and compared using independent-sample *t*-tests. Non-normally distributed variables were described as the median (interquartile range, IQR) and analyzed using nonparametric tests. Categorical variables were reported as numbers with percentages (n, %) and compared using the chi-square test or Fisher’s exact test, as appropriate. A two-sided *P* < 0.05 was considered statistically significant.

## Results

Between January 2022 and January 2025, 504 patients fulfilling the inclusion criteria were initially identified. Following exclusion of 4 patients with substantial missing clinical data (>30% of variables), 500 patients were included in the final analysis. Among these, 116 patients (23.2%) developed postoperative lower extremity DVT within the defined follow-up period, consistent with previously published incidence estimates for this population (2, 14, 15).

### Univariate analysis

Univariate analysis demonstrated significant between-group differences in the following variables (*P* < 0.05): age, Caprini score, preoperative D-dimer, lymphocyte count, and NLR (**Table 1**). No significant differences were identified for BMI, operative duration, tumor stage, or Histological type.

**Table 1:** Baseline characteristics of patients with and without lower extremity deep vein thrombosis (DVT) after gynecological malignant tumor surgery(n=500).

Variable	Non-DVT Group (N=384)	DVT Group (N=116)	t/W/χ^2^ value	P value
Histological type [n (%)]			0.142	0.931
Squamous-cell carcinoma	263 (68.5%)	80 (69.0%)		
Adenocarcinoma	87 (22.7%)	27 (23.3%)		
Other types	34 (8.9%)	9 (7.8%)		
Tumor Stage [n (%)]			5.322	0.150
Stage I	239 (62.2%)	61 (52.6%)		
Stage II	74 (19.3%)	29 (25.0%)		
Stage III	60 (15.6%)	19 (16.4%)		
Stage IV	11 (2.9%)	7 (6.0%)		
Comorbidities [n (%)]			2.150	0.143
No	297 (77.3%)	82 (70.7%)		
Yes	87 (22.7%)	34 (29.3%)		
Postoperative Bed Rest [n (%)]			3.193	0.203
< 3 days	111 (28.9%)	28 (24.1%)		
3–7 days	169 (44.0%)	62 (53.4%)		
> 7 days	104 (27.1%)	26 (22.4%)		
Age (years )	51.5±11.2	55.9±11.9	-3.690	<0.001
BMI(kg/m²)	24.9±3.9	25.4±3.8	-1.032	0.303
Operative Duration (h)	3.1±1.07	3.1±1.0	0.089	0.929
Intraoperative Blood Loss (ml)	100 (50–180)	100 (50–200)	19941.5	0.084
Caprini Score	3.4±1.5	4.6±2.7	-4.593	<0.001
Albumin (g/L)	41.7±5.5	40.1±6.0	2.660	0.008
Preoperative D-dimer (μg/L)	0.4 (0.26–0.88)	0.9 (0.4–1.7)	15222.0	<0.001
Fasting Blood Glucose (mmol/L)	5.6 ± 1.5	5.8 ± 1.8	-1.550	0.123
Platelet Count (×10^^9^/L)	289.5 ± 175.2	268.8 ± 200.2	1.076	0.283
Neutrophil Count (×10^^9^/L)	4.8 ± 3.3	4.6 ± 3.3	0.765	0.445
Lymphocyte Count (×10^^9^/L)	1.6 ± 0.7	1.1 ± 0.6	6.255	< 0.001
Monocyte Count (×10^^9^/L)	0.4 ± 0.2	0.4 ± 0.2	-0.575	0.565
APTT (s)	29.8 ± 3.2	29.3 ± 3.0	1.416	0.158
Thrombin Time (s)	14.1 ± 1.4	14.1 ± 1.0	0.187	0.852
Fibrinogen (g/L)	3.4 ± 1.3	3.4 ± 1.1	-0.601	0.548
NLR	3.9 ± 4.2	5.3 ± 3.9	-3.171	0.002
PLR	184.1 (138.0–247.0)	236.0 (180.3–363.6)	14585.0	< 0.001
LMR	3.9 ± 2.9	3.2 ± 1.9	2.387	0.017
MHR	0.4 ± 0.3	0.4 ± 0.2	1.048	0.296

Comorbidities included hypertension, diabetes mellitus, and coronary heart disease; BMI, body mass index; HDL-C, high-density lipoprotein cholesterol; APTT, activated partial thromboplastin time; NLR, neutrophil-to-lymphocyte ratio; PLR, platelet-to-lymphocyte ratio; LMR, lymphocyte-to-monocyte ratio; MHR, monocyte-to-high-density lipoprotein cholesterol ratio.

### Multivariable logistic regression

Variables with statistical significance in the univariate analysis were entered into the binary logistic regression model. The results revealed that age, Caprini score, preoperative D-dimer, lymphocyte count, and NLR were independent risk factors for postoperative lower-extremity DVT in patients with gynecologic tumors (P < 0.05), as detailed in **Table 2**.

**Table 2:** Univariable and multivariable logistic regression analyses of risk factors for postoperative DVT in patients with gynecological malignant tumors (Training set, N = 351).

Variable	Non-DVT Group (N=269)	DVT Group (N=82)	Univariable OR (95%CI, P)	Multivariable OR (95%CI, P)
Histological type[n (%)]				
Squamous-cell carcinoma	184 (68.4%)	50 (61%)		
Adenocarcinoma	62 (23%)	25 (30.5%)	1.48 (0.85-2.60, p=0.167)	
Other types	23 (8.6%)	7 (8.5%)	1.12 (0.45-2.76, p=0.805)	
Tumor Stage [n (%)]				
Stage I	179 (66.5%)	40 (48.8%)		
Stage II	46 (17.1%)	21 (25.6%)	2.04 (1.10-3.80, p=0.024)	1.48 (0.71-3.07, p=0.293)
Stage III	38 (14.1%)	16 (19.5%)	1.88 (0.96-3.71, p=0.067)	0.93 (0.40-2.15, p=0.865)
Stage IV	6 (2.2%)	5 (6.1%)	3.73 (1.08-12.83, p=0.037)	2.35 (0.61-9.12, p=0.216)
Postoperative Bed Rest [n (%)]				
< 3 days	84 (31.2%)	19 (23.2%)		
3–7 days	113 (42%)	43 (52.4%)	1.68 (0.91-3.09, p=0.094)	
> 7 days	72 (26.8%)	20 (24.4%)	1.23 (0.61-2.48, p=0.566)	
Comorbidities [n (%)]				
No	204 (75.8%)	57 (69.5%)		
Yes	65 (24.2%)	25 (30.5%)	1.38 (0.80-2.38, p=0.252)	
Age (years )	51.8 ± 11.1	55.8 ± 11.7	1.03 (1.01-1.06, p=0.006)	1.03(1.01-1.06, p=0.017)
BMI(kg/m²)	25.2 ± 3.9	25.5 ± 3.6	1.02 (0.96-1.09, p=0.450)	
Operative Duration (h)	3.1 ± 1.0	3.0 ± 1.0	0.92 (0.72-1.17, p=0.496)	
Intraoperative Blood Loss (ml)	100 (50-180)	100 (52.5-200)	1.00 (1.00-1.00, p=0.683)	
Caprini Score	3.4 ± 1.5	4.5 ± 3.0	1.29 (1.14-1.45, p<0.001)	1.31 (1.14-1.50, p<0.001)
Albumin (g/L)	41.3 ± 5.6	40.5 ± 6.4	0.97 (0.93-1.02, p=0.229)	
Preoperative D-dimer (μg/L)	0.4 (0.3-0.9)	0.7 (0.4-1.6)	1.32 (1.11-1.55, p=0.001)	1.27 (1.06-1.52, p=0.010)
Fasting Blood Glucose (mmol/L)	5.5 ± 1.5	6.1 ± 2.0	1.18 (1.03-1.35, p=0.020)	1.12 (0.97-1.31, p=0.132)
Platelet Count (×10^9/L)	294.2 ± 193.7	254.1 ± 182.1	1.00 (1.00-1.00, p=0.103)	
Neutrophil Count (×10^9/L)	4.9 ± 3.5	4.4 ± 3.1	0.95 (0.88-1.03, p=0.210)	
Lymphocyte Count (×10^9/L)	1.5 ± 0.7	1.0 ± 0.6	0.24 (0.15-0.41, p<0.001)	0.25 (0.12-0.51, p<0.001)
Monocyte Count (×10^9/L)	0.4 ± 0.2	0.4 ± 0.2	1.03 (0.33-3.19, p=.957)	
APTT (s)	29.5 ± 3.1	29.3 ± 2.9	0.98 (0.90-1.06, p=0.546)	
Thrombin Time (s)	14.1 ± 1.3	14.1 ± 1.0	0.99 (0.81-1.22, p=0.954)	
Fibrinogen (g/L)	3.4 ± 1.4	3.5 ± 1.1	1.06 (0.90-1.25, p=0.489)	
NLR	4.0 ± 4.0	5.4 ± 3.9	1.08 (1.02-1.14, p=0.009)	0.91 (0.84-0.99, p=0.035)
PLR	189.6(138.4-249.7)	257.4 (180.9-370.8)	1.01 (1.00-1.01, p<0.001)	1.00 (1.00-1.01, p=0.050)
LMR	3.9 ± 3.1	3.2 ± 1.9	0.88 (0.78-1.00, p=0.048)	1.00 (0.90-1.11, p=0.992)
MHR	294.2 ± 193.7	254.1 ± 182.1	1.00 (1.00-1.00, p=0.103)	

Comorbidities included hypertension, diabetes mellitus, and coronary heart disease; BMI, body mass index; APTT, activated partial thromboplastin time; NLR, neutrophil-to-lymphocyte ratio; PLR, platelet-to-lymphocyte ratio; LMR, lymphocyte-to-monocyte ratio; MHR, monocyte-to-high-density lipoprotein cholesterol ratio.

### Model development and performance

In the validation set, GBM achieved the highest AUC value (0.840, 95% CI 0.767–0.906), while CatBoost yielded an AUC of 0.816 (95% CI 0.738–0.881). The DeLong test confirmed a statistically significant difference in AUC between the two models (P < 0.05), indicating that GBM possessed better discriminative ability. Nevertheless, considering the balanced comprehensive performance and lower overfitting risk in clinical scenarios, CatBoost was finally determined as the candidate prediction model. The performance indicators of CatBoost were as follows: AUC = 0.816 (95% CI: 0.738–0.881), sensitivity = 0.824 (95% CI: 0.700–1.000), specificity = 0.730 (95% CI: 0.515–0.876), precision = 0.475 (95% CI: 0.337–0.675), and F1-score = 0.602 (95% CI: 0.494–0.728). Detailed results are presented in **Table 3** and **Figure 1**. Calibration and decision curve analyses confirmed its acceptable calibration and potential clinical utility (**Figures 2**, **3**).

**Table 3:** Comparison of 10 Machine Learning Model Performance Metrics.

Model	Threshold	AUC (95%CI)	Accuracy (95%CI)	Sensitivity (95%CI)	Specificity (95%CI)	Precision (95%CI)	F1 (95%CI)
Logistic	0.180	0.775 (0.686-0.848)	0.691 (0.604-0.819)	0.853 (0.621-0.971)	0.643 (0.513-0.852)	0.414 (0.318-0.609)	0.558 (0.462-0.688)
SVM	0.218	0.79 (0.692-0.875)	0.732 (0.654-0.812)	0.882 (0.761-1.000)	0.687 (0.589-0.787)	0.455 (0.341-0.589)	0.600 (0.486-0.718)
GBM	0.224	0.84 (0.767-0.906)	0.792 (0.651-0.859)	0.824 (0.740-1.000)	0.783 (0.561-0.864)	0.528 (0.367-0.667)	0.644 (0.524-0.752)
Neural Network	0.177	0.773 (0.69-0.848)	0.685 (0.607-0.816)	0.882 (0.667-0.974)	0.626 (0.529-0.836)	0.411 (0.311-0.591)	0.561 (0.463-0.692)
Random Forest	0.269	0.797 (0.718-0.873)	0.765 (0.594-0.839)	0.735 (0.600-0.967)	0.774 (0.487-0.864)	0.49 (0.327-0.647)	0.588 (0.466-0.71)
Xgboost	0.497	0.807 (0.725-0.873)	0.698 (0.591-0.819)	0.853 (0.695-1.000)	0.652 (0.482-0.831)	0.42 (0.304-0.595)	0.563 (0.460-0.689)
KNN	0.181	0.738 (0.635-0.824)	0.685 (0.456-0.849)	0.706 (0.462-1.000)	0.678 (0.316-0.921)	0.393 (0.261-0.678)	0.505 (0.400-0.657)
Adaboost	0.144	0.629 (0.535-0.718)	0.758 (0.691-0.832)	0.382 (0.160-0.538)	0.87 (0.804-0.964)	0.464 (0.29-0.681)	0.419 (0.215-0.562)
LightGBM	0.024	0.722 (0.619-0.82)	0.765 (0.698-0.839)	0.794 (0.639-0.929)	0.757 (0.676-0.838)	0.491 (0.364-0.626)	0.607 (0.484-0.712)
CatBoost	0.550	0.816 (0.738-0.881)	0.752 (0.617-0.852)	0.824 (0.700-1.000)	0.73 (0.515-0.876)	0.475 (0.337-0.675)	0.602 (0.494-0.728)

Logistic,Logistic Regression; SVM,Support Vector Machine;GBM,Gradient Boosting Machine;XGBoost,Extreme Gradient Boosting; KNN, k-Nearest Neighbors; AdaBoost, Adaptive Boosting; LightGBM,Light Gradient Boosting Machine; CatBoost,Categorical Boosting; F1, The harmonic mean of precision and sensitivity.The optimal threshold for each model was determined by the Youden index (maximizing sensitivity + specificity - 1).

**Figure 1:**
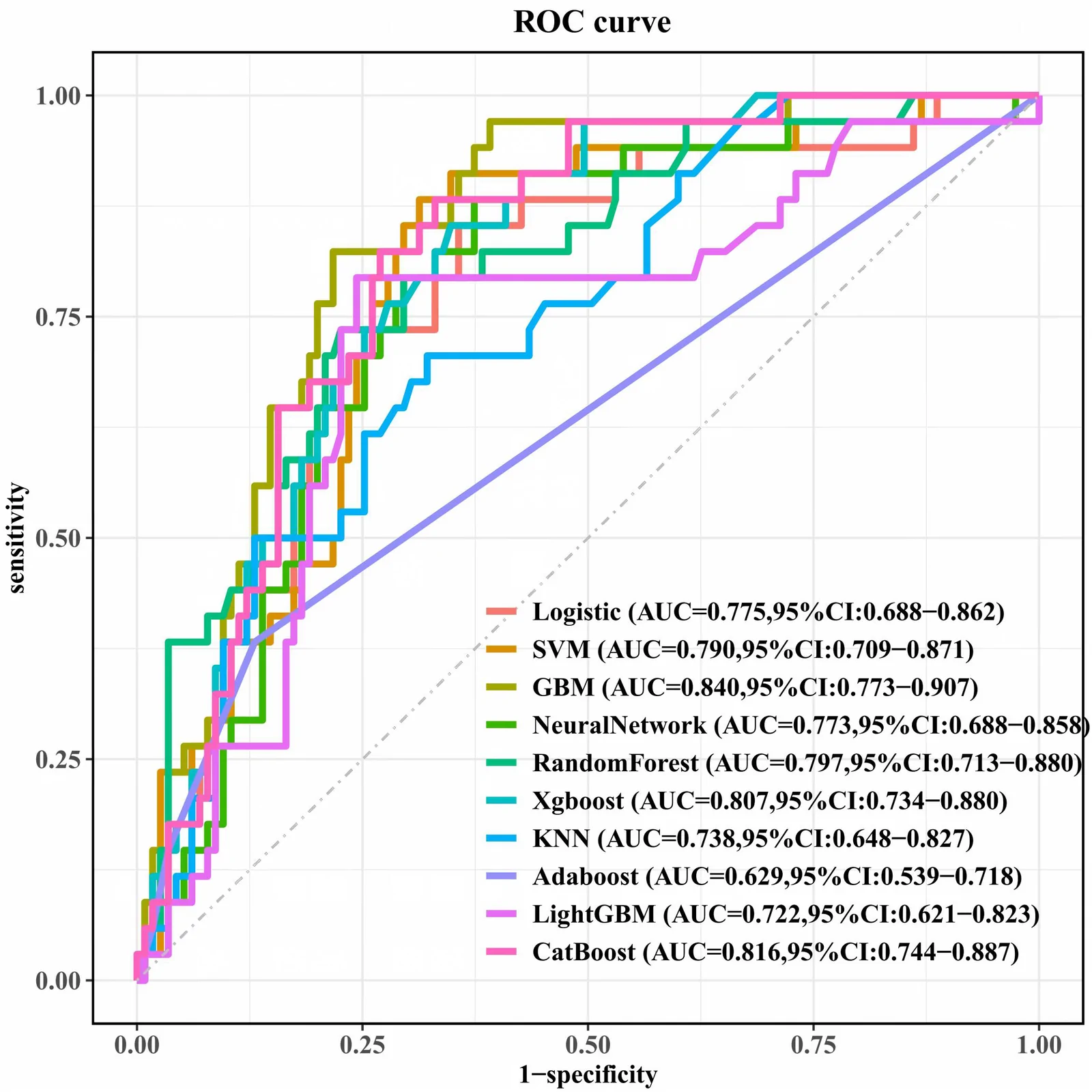
Receiver operating characteristic (ROC) curves and area under the curve (AUC) for ten machine learning models on the validation set.

**Figure 2:**
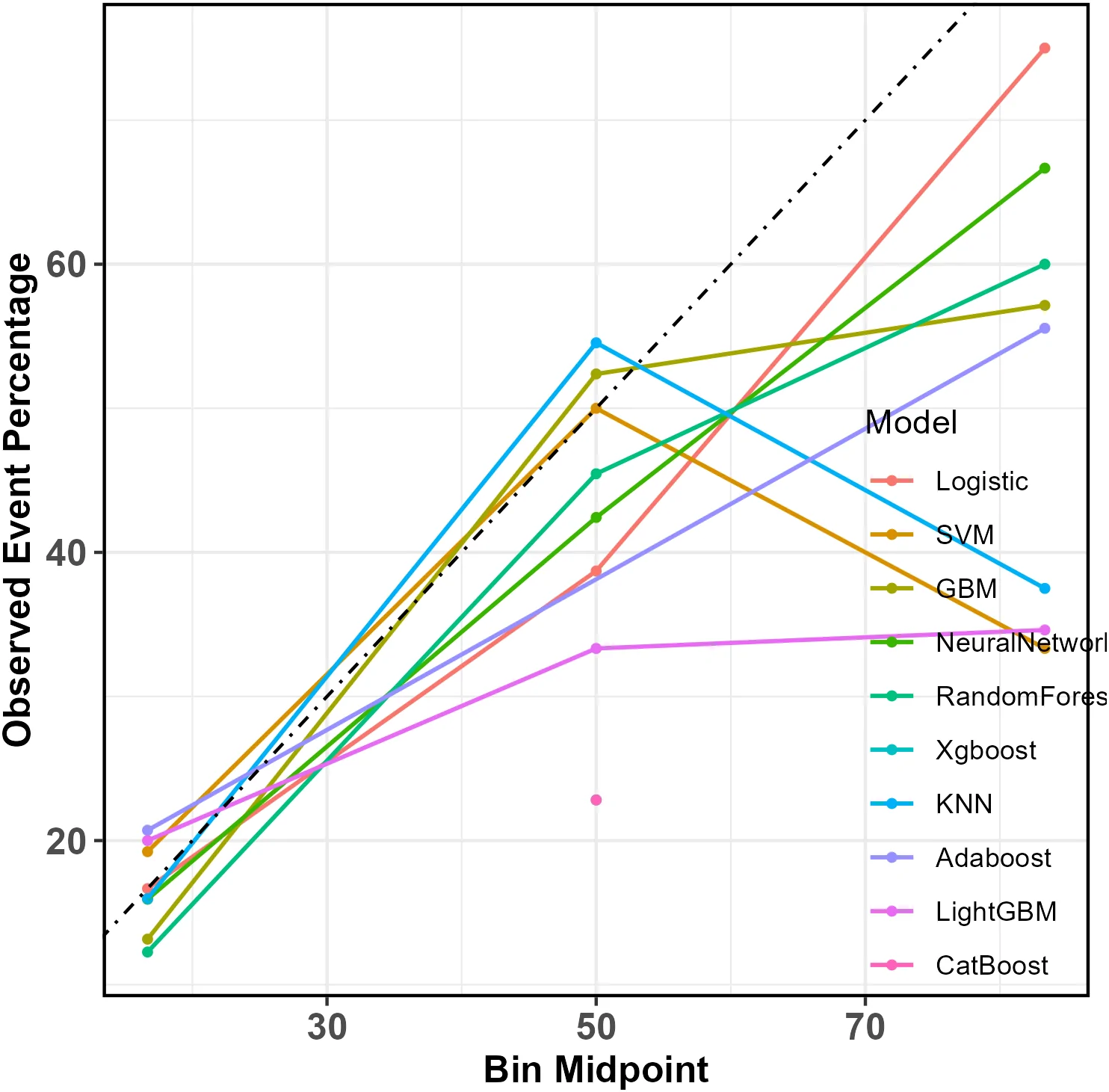
Calibration curves of ten machine learning models on the validation set.

**Figure 3:**
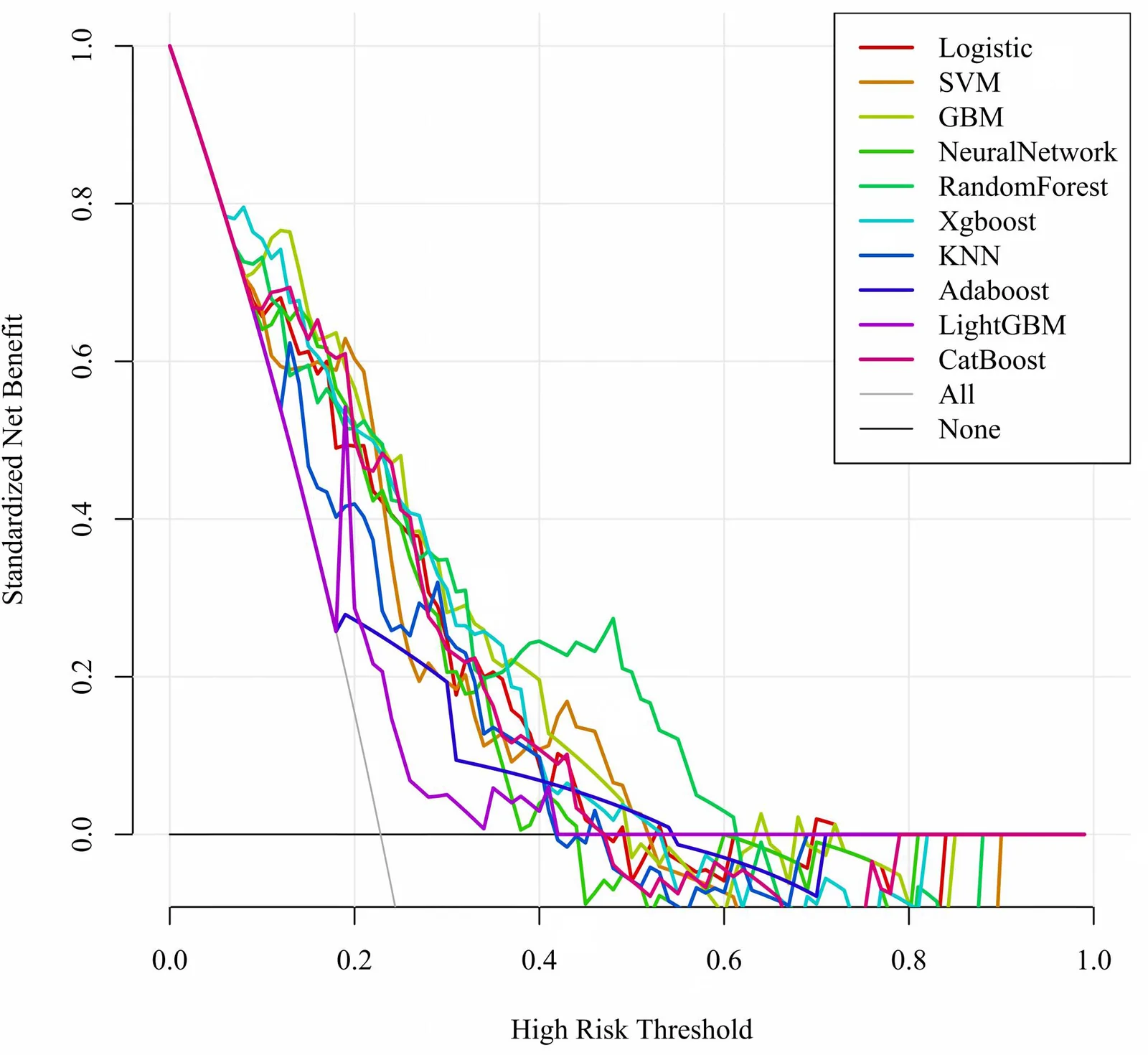
Decision curve analysis (DCA) of ten machine learning models on the validation set.

### Model interpretation

To enhance interpretability of the CatBoost model, we applied SHAP analysis (**Figures 4**–**8**). Ranking features by their mean absolute SHAP values identified lymphocyte count as the strongest predictor of postoperative DVT, followed by Caprini score, age, preoperative D-dimer, and NLR.The SHAP summary plot confirmed that higher Caprini score, older age, elevated preoperative D-dimer, and lower lymphocyte count were associated with increased risk predictions. Force plots of two representative patients provided clear, case-specific explanations: for a true-positive patient, the risk prediction was increased by multiple adverse factors, while for a true-negative patient, protective factors dominated, lowering the predicted probability. These visualizations demonstrate the model’s transparency at the individual patient level.

**Figure 4:**
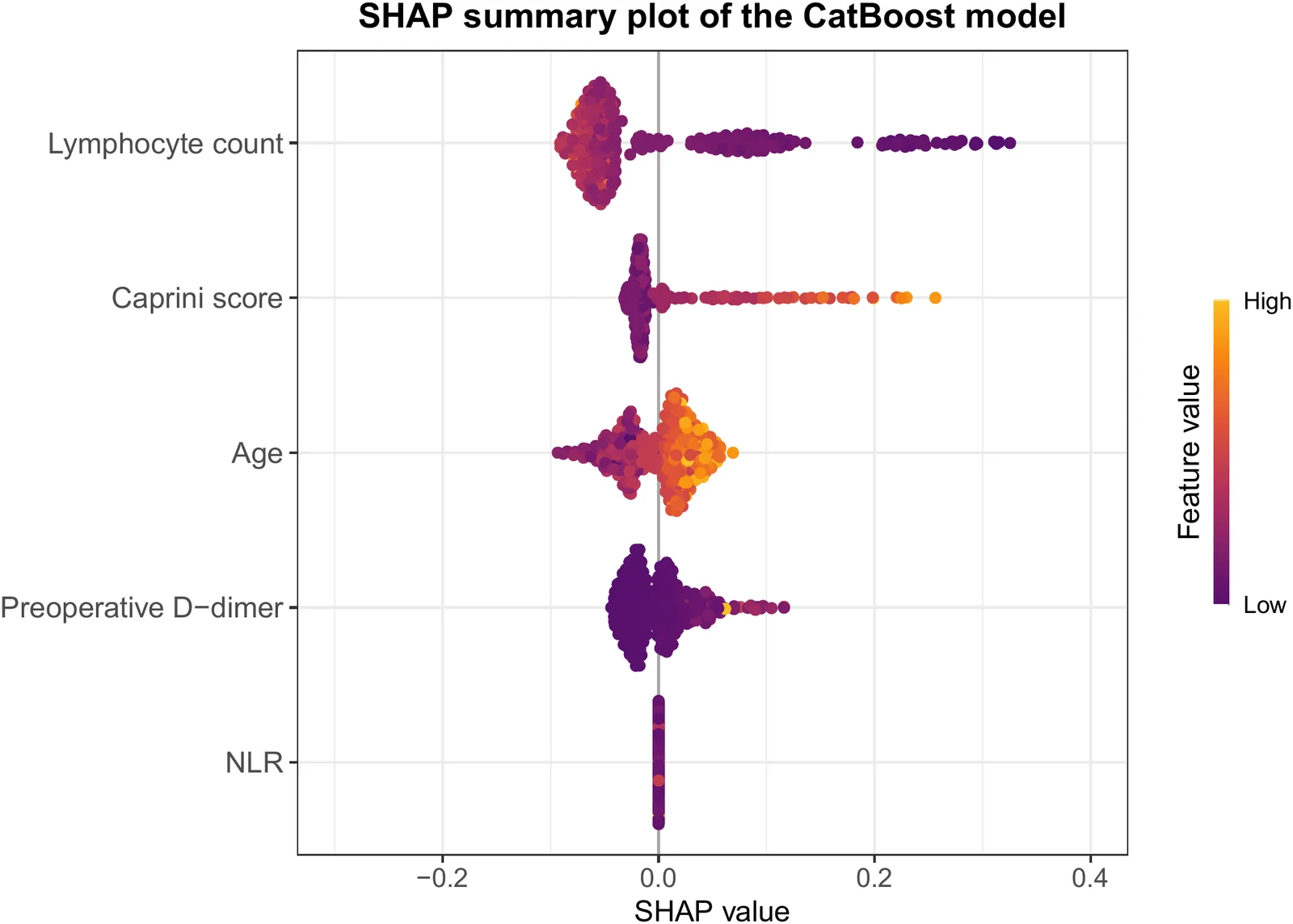
SHAP summary plot (beeswarm plot) for the CatBoost model.The horizontal position of each dot corresponds to the SHAP value, reflecting the direction and magnitude of each feature’s effect on the predicted risk of postoperative deep-vein thrombosis. Feature values are color-coded: purple denotes low feature values, and orange-yellow denotes high feature values.

**Figure 5:**
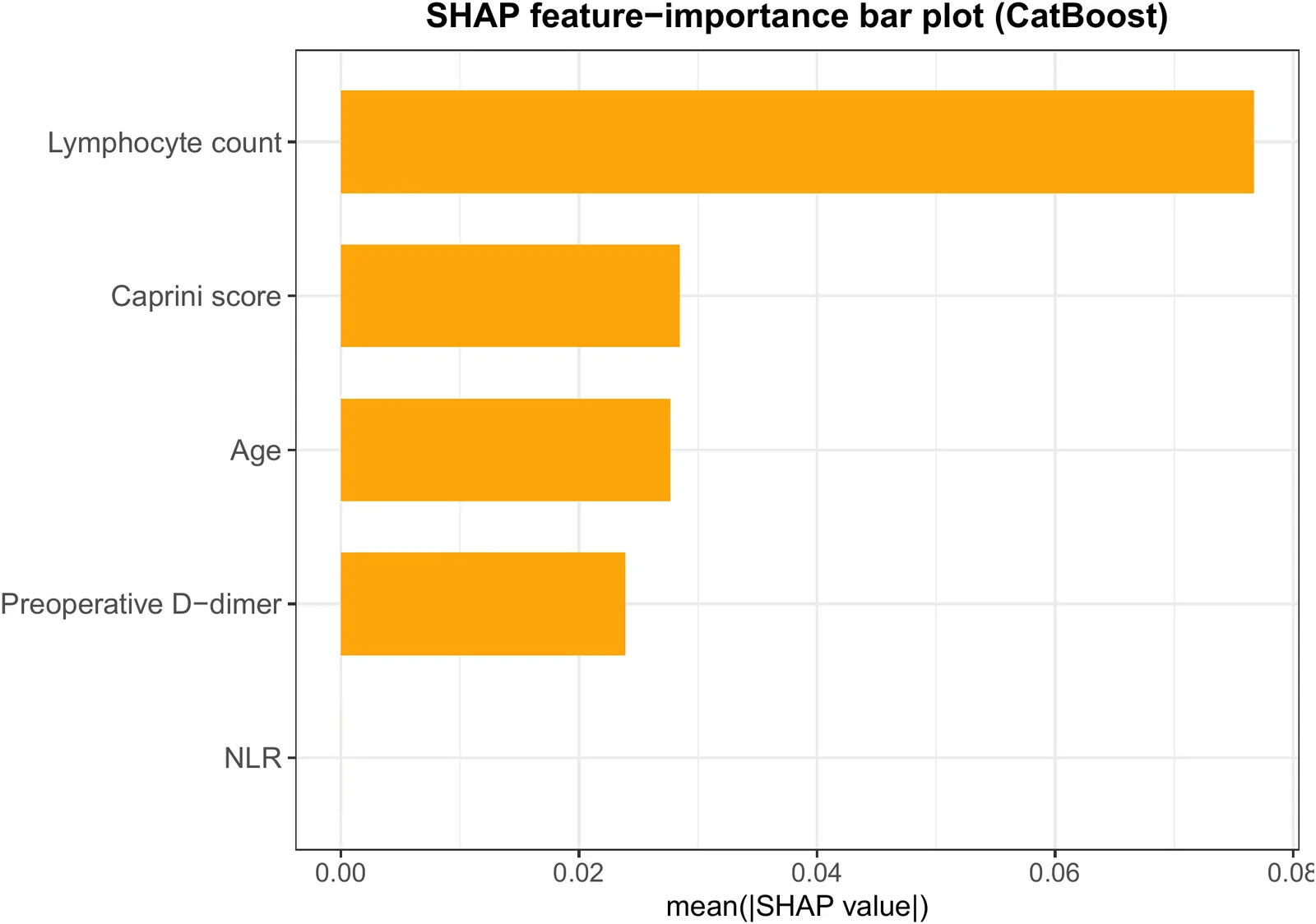
SHAP feature importance bar plot for the CatBoost model (ranked by mean absolute SHAP value). The horizontal axis represents the mean absolute SHAP value, and the length of each bar indicates the average predictive contribution of each variable to the model for postoperative deep-vein thrombosis risk. Variables are ranked according to their mean absolute SHAP values.

**Figure 6:**
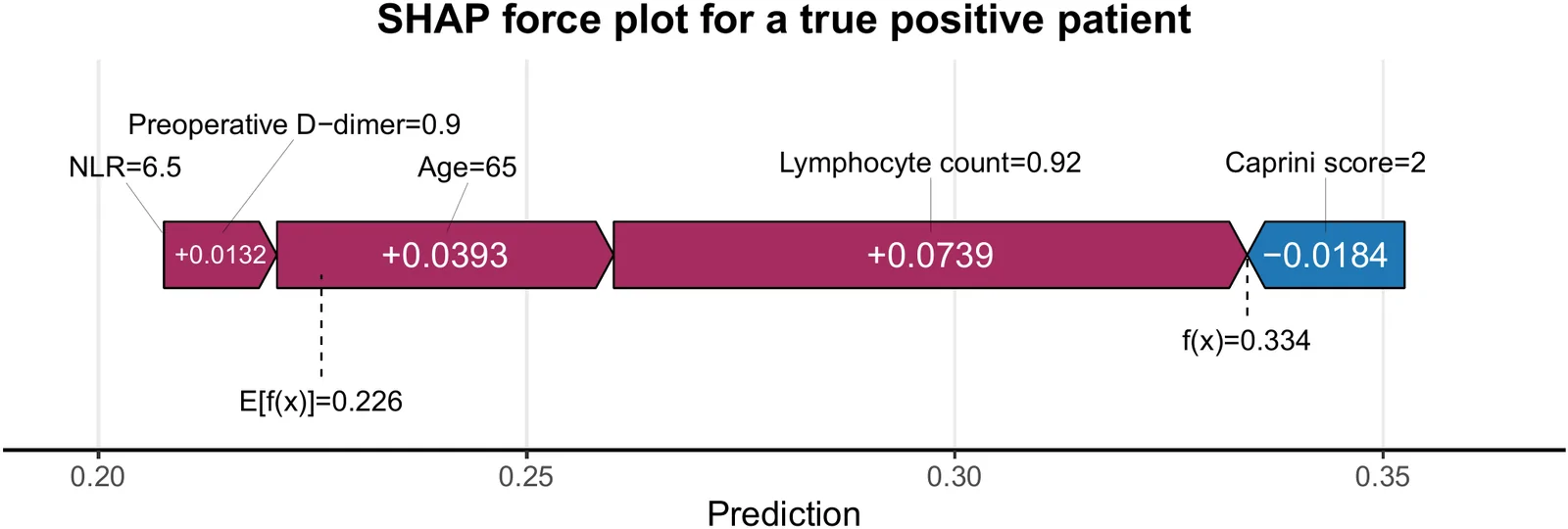
SHAP force plot of true positive patient prediction with the CatBoost model.Red segments represent features that increase the predicted risk of postoperative deep-vein thrombosis (positive SHAP values), while the blue segment represents a feature decreasing the predicted risk (negative SHAP value). E[f(x)] denotes the baseline expected model prediction across all samples, and f(x) is the final predicted risk for this individual patient.

**Figure 7:**
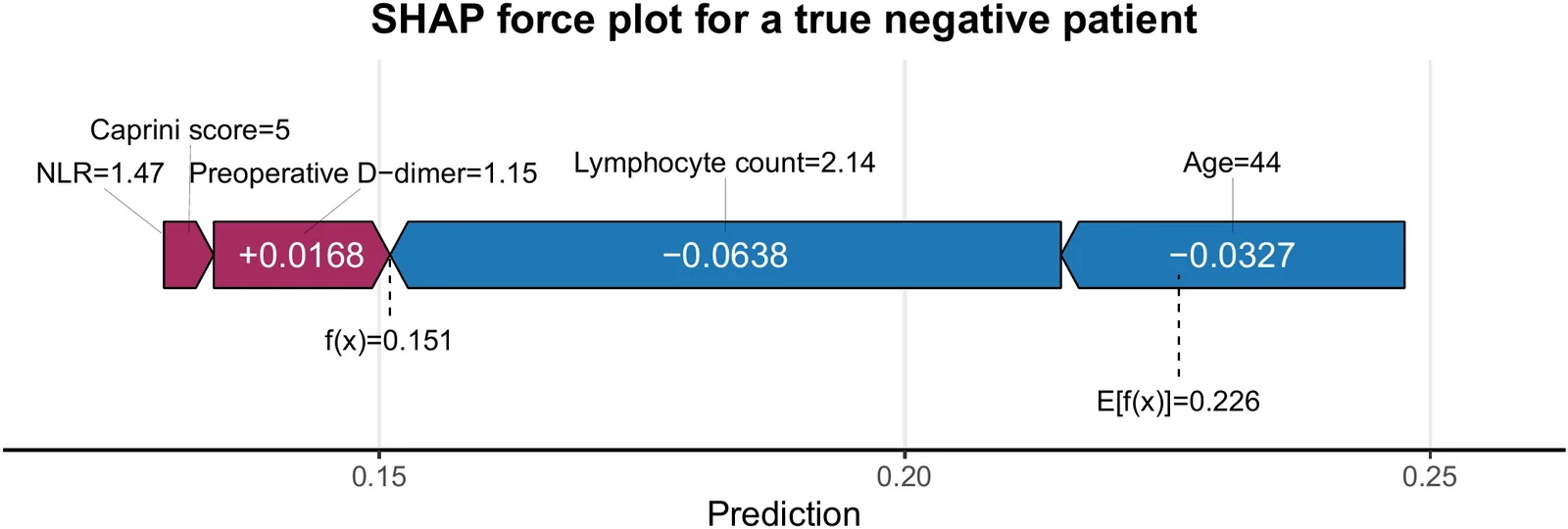
SHAP force plot of true negative patient prediction with the CatBoost model.Notations are consistent with **Figure 6**.

**Figure 8:**
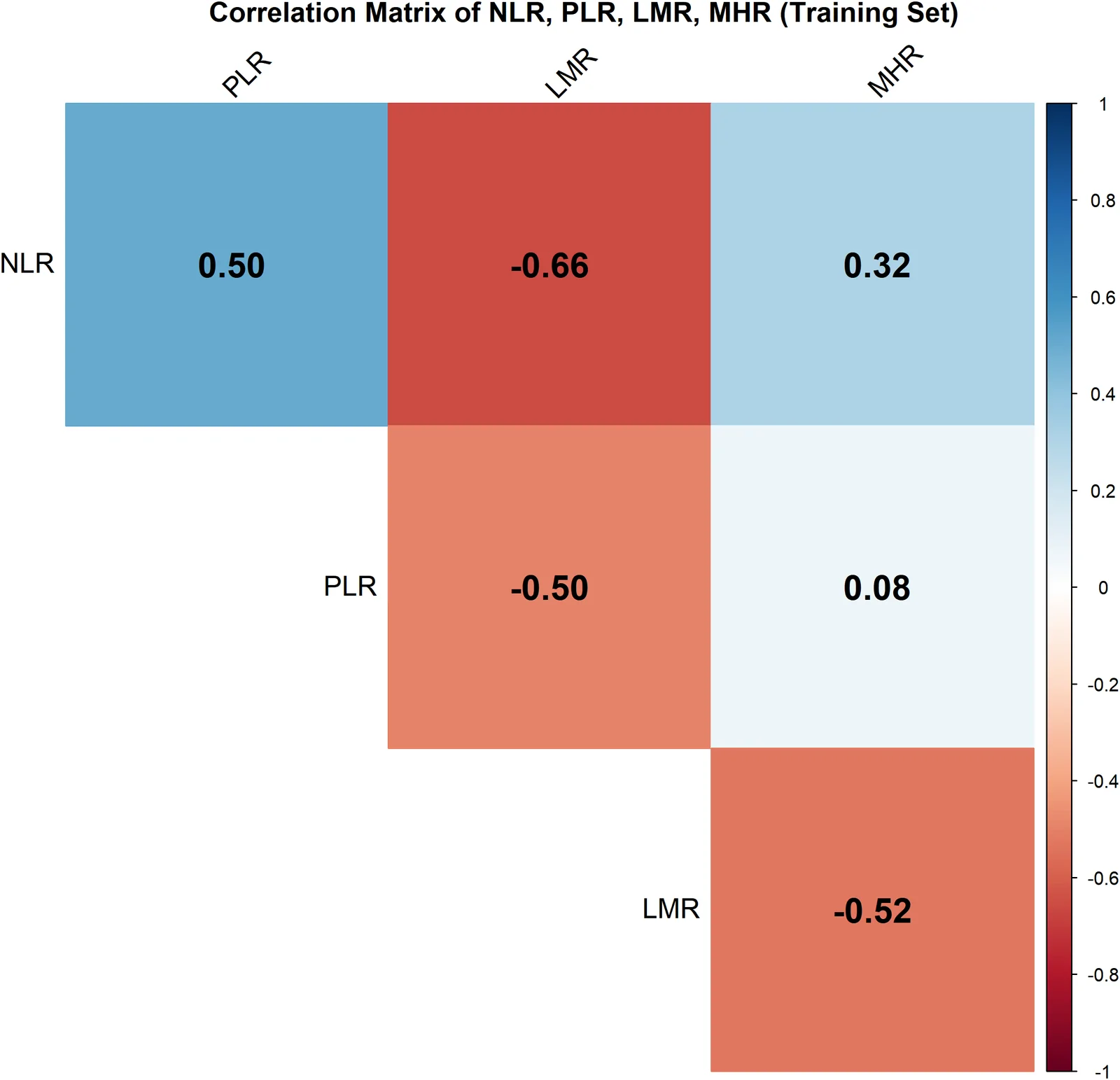
Correlation matrix of NLR, PLR, LMR, and MHR in the training set.

## Discussion

### High incidence of postoperative DVT in gynecological malignancy patients

The present study documented a postoperative lower extremity DVT incidence of 23.2% in gynecological malignancy patients following radical surgery, consistent with previously reported rates of 19.6%~38% (2) and corroborating the findings of Lin B (14) and Li YJ et al. (15) The substantially elevated DVT risk in this population is attributable to the pathophysiological convergence of Virchow’s triad: (1) tumor-induced hypercoagulability mediated by cancer procoagulant release and aberrant tissue factor expression; (2) vascular endothelial injury resulting from extensive pelvic surgical dissection and pneumoperitoneum-related inferior vena cava compression during laparoscopic procedures; and (3) venous stasis secondary to intraoperative lithotomy positioning constraints and prolonged postoperative immobility. Compounding patient-level risk factors, including advanced age, obesity, and systemic comorbidities, further amplify thrombotic susceptibility.

Among gynecologic malignancies, ovarian cancer carries a particularly high thrombotic risk. Large pelvic tumors and malignant ascites in advanced disease cause extrinsic compression of pelvic and iliac veins, impairing venous outflow and inducing local stasis to promote thrombosis. Together with tumor derived procoagulants and inflammatory cytokines, such mechanical compression synergistically elevates susceptibility to venous thromboembolism in ovarian cancer patients. A meta-analysis by Weeks et al (16). demonstrated that patients with stage III-IV ovarian cancer had a higher VTE risk than those with early stage disease (OR = 2.73, 95%CI:1.84-4.06), and ascites was also associated with elevated VTE risk (OR = 2.12, 95%CI:1.51-2.96) Another meta-analysis conducted by Xu et al (17). focusing on postoperative epithelial ovarian cancer patients further validated that advanced FIGO stage and ascites were important risk factors for postoperative venous thromboembolism, with a pooled OR of 2.07 for ascites. In their report, the pooled incidence of symptomatic postoperative VTE was 3%, and the overall incidence including asymptomatic subclinical VTE reached 8%. Consistent findings from these two meta-analyses indicate that advanced tumor stage and ascites contribute to increased thrombotic risk via multiple mechanisms including cancer-related hypercoagulability and mechanical compression of pelvic vessels.

### CatBoost as the selected candidate predictive algorithm

Among the ten ML algorithms evaluated, CatBoost exhibited well-balanced overall performance in the independent validation set (AUC = 0.816, sensitivity = 0.824, specificity = 0.730), although GBM yielded a statistically higher AUC. CatBoost is a gradient boosting framework that employs ordered boosting and symmetric decision trees, with native handling of categorical features without requiring extensive preprocessing. These architectural advantages may confer particular suitability for clinical datasets characterized by mixed data types (continuous laboratory parameters alongside categorical variables such as anticoagulation status and comorbidities) and moderate sample sizes. The high sensitivity of the CatBoost model (0.824) is of particular clinical significance in this context, as missed diagnoses of DVT carry substantial consequences in terms of PE risk and associated morbidity.

To address the inherent “black box” opacity of ensemble tree methods, SHAP analysis-a unified framework grounded in Shapley values from cooperative game theory — was applied to decompose predictions into individual feature contributions. SHAP provides both global feature importance rankings and patient-specific prediction decompositions, thereby bridging the gap between algorithmic precision and clinical interpretability. This dual capacity is essential for building clinician trust in AI-assisted decision tools and facilitating evidence-informed shared decision-making.

### Clinical implications of key SHAP-identified predictors

SHAP analysis demonstrated that lymphocyte count had the greatest predictive contribution to postoperative deep vein thrombosis in the CatBoost model established in this study. This innovative finding differs from most previous studies, which usually regarded D-dimer, Caprini score, or inflammatory markers such as NLR as core predictors (18–20). Current research on postoperative thrombosis has mainly focused on coagulation markers, traditional clinical scores, and inflammatory indicators, while few studies have identified lymphocyte count as the primary predictor.

Malignant tumors can induce chronic inflammation, immunosuppression, and hypercoagulability, which interact to form the pathological basis of cancer-associated thrombosis (21, 22). As the core cells of adaptive immunity, decreased lymphocyte count impairs vascular endothelial repair, promotes coagulation activation, disrupts the coagulation-fibrinolysis balance, and increases thrombotic risk. In gynecologic malignancies, the tumor microenvironment and surgical stress further accelerate lymphocyte apoptosis and aggravate immune dysfunction (23).

Notably, previous machine learning studies mostly included general surgical or early-stage tumor patients, whereas this study mainly enrolled middle-advanced patients with more severe immunosuppression, which may explain why lymphopenia showed higher predictive value than traditional markers. This discrepancy suggests that thrombosis prediction models have obvious population specificity. Immune-related indicators have unique value in cancer patients, and combined assessment of immune, coagulation, and clinical indicators can improve predictive accuracy. Future prospective studies are warranted to incorporate lymphocyte count into tumor-specific thrombosis risk scores to guide individualized thromboprophylaxis.

As the most widely applied risk assessment tool for venous thromboembolism, the validity of the Caprini score has been extensively validated in clinical research (24–26). In the present study, the Caprini score ranked second in variable importance, consistent with previous literature (27, 28), further confirming its stable predictive value in postoperative surgical patients. The Caprini score integrates multiple risk factors including age, operative duration, malignancy, mobility, and comorbidities, reflecting thrombotic risk from a holistic clinical perspective. Nevertheless, its importance was lower than that of lymphocyte count in our model, indicating that conventional clinical scores have limited capacity to reflect underlying pathological states such as immune and inflammatory disturbances. Therefore, a combined assessment strategy of “Caprini score + lymphocyte count + D-dimer” can be adopted in clinical practice, which not only accounts for traditional risk factors but also reflects immune and coagulation status, thereby improving the accuracy of risk stratification.

Age is a well-established independent risk factor for venous thrombosis. In this study, age ranked third, which is consistent with epidemiological and machine learning studies worldwide (29–31). With advancing age, reduced vascular compliance, sluggish blood flow, higher prevalence of cardiovascular diseases and diabetes mellitus, as well as prolonged bed rest and decreased mobility after surgery, collectively contribute to an elevated thrombotic risk. The stable predictive role of age suggests that advanced age represents an indispensable fundamental factor in thrombosis assessment regardless of the modeling approach.

As a classic biomarker reflecting systemic activation of coagulation and fibrinolysis, the predictive value of preoperative D-dimer has been widely acknowledged (32, 33). However, its relative importance was lower than that of lymphocyte count and Caprini score in our study, which may be explained by the non-specific elevation of D-dimer induced by malignancy itself, reducing its specificity for postoperative thrombosis. This finding also implies that in cancer populations, D-dimer is more suitable for ruling out thrombosis rather than serving as an isolated high-risk predictive marker.

NLR exhibited a relatively low contribution in the present model. Although numerous studies have suggested that NLR reflects systemic inflammatory-immune imbalance and correlates with thrombotic risk (34, 35), its predictive performance was limited in our study, which may be attributed to two factors. First, strong collinearity exists between NLR and lymphocyte count; after incorporating lymphocytes into the model, NLR provides little additional predictive information. Second, the study population was mainly characterized by lymphopenia without prominent neutrophilia, resulting in a narrow range of NLR variations and attenuated predictive efficacy. This also indicates that not all inflammatory markers are universally applicable across populations, and indicator selection should be tailored to specific disease characteristics.

In summary, the CatBoost model established in this study shows favorable predictive performance and interpretability. To our knowledge, this is the first study to confirm that lymphocyte count serves as the strongest predictor of postoperative thrombosis in patients with gynecologic malignancies, addressing the insufficient attention paid to immune markers in existing literature. In future clinical practice, preoperative lymphocyte count can be recommended as a routine screening index, and intensive thromboprophylaxis should be initiated early in patients with low lymphocyte counts. Further large sample studies with long term follow up are encouraged to verify the causal relationship between immune markers and tumor associated thrombosis, and to develop prediction models more tailored to cancer patients.

### Limitations and future directions

Several limitations of this study merit acknowledgment. First, the retrospective, single-center design at a tertiary cancer center may restrict the generalizability of findings to institutions with different case-mix profiles or thromboprophylaxis practices. Second, the moderate sample size (n = 500), while adequate for initial model development, may constrain robustness under subgroup analyses and limit the detection of smaller effect sizes. Third, the prediction model requires prospective external validation before clinical implementation. Fourth, this study used a fully de-identified retrospective dataset, so original medical records were unavailable. Owing to the lack of primary tumor site data, variables such as pelvic mass and malignant ascites could not be extracted, and subgroup analyses for specific tumors including ovarian cancer were infeasible. Furthermore, despite hospital-wide standardized thromboprophylaxis protocols, individual level implementation details (anticoagulant dosage, prophylaxis duration, timing of postoperative duplex ultrasound, differentiation between symptomatic and screening detected DVT) could not be obtained from the dataset.

Future research priorities should encompass: (1) multi-center prospective external validation in geographically and clinically diverse cohorts to mitigate limitations related to single-center recruitment and improve generalizability; (2) expansion of sample size to enable robust subgroup analyses across different primary tumor sites; (3) prospective clinical evaluation of this predictive model before real-world bedside application; (4) collection of complete source medical record data including pelvic mass size, malignant ascites status, detailed perioperative thromboprophylaxis information, and distinction between symptomatic and screening-detected DVT, which will facilitate deeper mechanistic exploration and stratified risk assessment. The development of a web-based risk calculator incorporating the CatBoost model is under consideration as a subsequent translational objective.

## Conclusion

This study developed and validated an interpretable CatBoost-based machine learning prediction model for postoperative lower extremity DVT in gynecological malignancy patients, demonstrating strong discriminatory performance (AUC = 0.816) and clinically meaningful sensitivity (0.824) on an independent validation cohort. SHAP analysis revealed lymphocyte count, Caprini score, age, preoperative D-dimer, and neutrophil-to-lymphocyte ratio (NLR) as the five most influential predictive features, providing mechanistically grounded and actionable insights for clinical practice. The integration of interpretable AI into perioperative DVT risk assessment offers a promising pathway to precision thromboprophylaxis, individualized nursing care, and ultimately improved outcomes in this high-risk population.

## Data Availability

The original contributions presented in the study are included in the article/**Supplementary Material**. Further inquiries can be directed to the corresponding authors.
