# Supplementary material for: Explainable machine-learning model for postoperative lower-extremity deep vein thrombosis after gynecologic cancer surgery
Source: Front Oncol. 2026 Sep 30;16:1813354. doi: 10.3389/fonc.2026.1813354 (PMC13661352; doi:10.3389/fonc.2026.1813354)
Supplement: Supplementary file 1 [file Table1.doc]

Supplementary Table S1. Hyper-parameter tuning results of different models

| Model | Parameter range | Optimal parameters |
| --- | --- | --- |
| Logistic | None | None |
| SVM | sigma = c(0.1,0.01,0.001);C = seq(0.01,0.1,by=0.01) | sigma = 0.001, C = 0.09 |
| GBM | n.trees = seq(100,1000,by=100),interaction.depth = seq(1,5,by=1) | n.trees = 100, interaction.depth = 5,shrinkage = 0.1, n.minobsinnode = 30 |
| NeuralNetwork | size = seq(1,10,by=1),decay = seq(0.1,1,by=0.1) | size = 6,decay = 0.6 |
| RandomForest | mtry = seq(2,15,by=1),numRandomCuts = seq(1,10,by=1) | mtry = 11,numRandomCuts = 3 |
| Xgboost | max_depth = seq(1,5,by=1),eta = c(0.1,0.01,0.001),gamma = c(0.1,0.5,0.1),colsample_bytree = c(0.1,1,1),min_child_weight = c(0.1,1,1),subsample = c(0.1,1,1) | max_depth = 3,eta = 0.001,gamma = 0.5,colsample_bytree = 0.5,min_child_weight = 1,subsample = 0.6 |
| KNN | kmax = seq(1,15,by=1) ,distance = c(1,2),kernel = "optimal" | kmax = 12 ,distance = 1,kernel = "optimal" |
| Adaboost | mfinal = seq(2,10,by=1),maxdepth = seq(1,5,by=1),coeflearn = "Zhu" | mfinal = 2,maxdepth = 2,coeflearn = "Zhu" |
| LightGBM | objective = "binary",metric = "auc",min_data = seq(1,10,by=1),learning_rate = seq(0.1,1,by=0.1), num_threads = seq(1,10,by=1) | objective = "binary",metric = "auc",min_data = 1,learning_rate = 1.0, num_threads = 2 |
| CatBoost | ignored_features = c(4:9),border_count = (8,32,64,128),depth = seq(1,5,by=1),learning_rate = seq(0.01,0.1,by=0.01) | ignored_features = 5,border_count = 32,depth = 5,learning_rate = 0.03 |
