# Supplementary material for: Explainable machine-learning model for postoperative lower-extremity deep vein thrombosis after gynecologic cancer surgery
Source: Front Oncol. 2026 Sep 30;16:1813354. doi: 10.3389/fonc.2026.1813354 (PMC13661352; doi:10.3389/fonc.2026.1813354)
Supplement: Supplementary file 2 [file Table2.doc]

Supplementary Table S2. C-index and Brier score of each model in the test set

| Model | Brier score | cindex |
| --- | --- | --- |
| Logistic | 0.15（0.11-0.19） | 0.77（0.76-0.79） |
| SVM | 0.16（0.12-0.19） | 0.79（0.78-0.80） |
| GBM | 0.14（0.10-0.17） | 0.84（0.83-0.85） |
| NeuralNetwork | 0.15（0.12-0.19） | 0.77（0.760.79） |
| RandomForest | 0.14（0.11-0.18） | 0.80（0.78-0.81） |
| Xgboost | 0.25（0.25-0.26） | 0.81（0.80-0.82） |
| KNN | 0.16（0.12-0.21） | 0.74（0.72-0.75） |
| Adaboost | 0.20（0.14-0.26） | 0.63（0.61-0.64） |
| LightGBM | 0.24（0.18-0.31） | 0.72（0.71-0.74） |
| CatBoost | 0.27（0.26-0.28） | 0.82（0.80-0.83） |
