# Supplementary material for: Explainable machine-learning model for postoperative lower-extremity deep vein thrombosis after gynecologic cancer surgery
Source: Front Oncol. 2026 Sep 30;16:1813354. doi: 10.3389/fonc.2026.1813354 (PMC13661352; doi:10.3389/fonc.2026.1813354)
Supplement: Supplementary file 3 [file Table3.doc]

Supplementary Table S3. DeLong test results between CatBoost and other models

| Model 1 | Model 2 | *Z* value | *P* value |
| --- | --- | --- | --- |
| CatBoost | Logistic | 3.755 | <0.001 |
| CatBoost | SVM | 4.316 | <0.001 |
| CatBoost | GBM | -2.858 | 0.004 |
| CatBoost | NeuralNetwork | 3.419 | 0.001 |
| CatBoost | RandomForest | -5.575 | <0.001 |
| CatBoost | Xgboost | 1.606 | 0.05 |
| CatBoost | KNN | -4.337 | <0.001 |
| CatBoost | Adaboost | 6.657 | <0.001 |
| CatBoost | LightGBM | -5.575 | <0.001 |
